# Deconstructing Neurogenesis, Transplantation and Genome-Editing as Neural Repair Strategies in Brain Disease

**DOI:** 10.3389/fcell.2020.00116

**Published:** 2020-03-13

**Authors:** Muhammad O. Chohan

**Affiliations:** ^1^Department of Psychiatry, Division of Integrative Neuroscience, New York State Psychiatric Institute, New York, NY, United States; ^2^Department of Psychiatry, Division of Child and Adolescent Psychiatry, Columbia University, New York, NY, United States

**Keywords:** neural stem cells, transplantation, adult neurogenesis, neurological disorders, genome-editing

## Abstract

Neural repair in injury and disease presents a pressing unmet need in regenerative medicine. Due to the intrinsically reduced ability of the brain to replace lost and damaged neurons, reversing long-term cognitive and functional impairments poses a unique problem. Over the years, advancements in cellular and molecular understanding of neurogenesis mechanisms coupled with sophistication of biotechnology tools have transformed neural repair into a cross-disciplinary field that integrates discoveries from developmental neurobiology, transplantation and tissue engineering to design disease- and patient-specific remedies aimed at boosting either native rehabilitation or delivering exogenous hypoimmunogenic interventions. Advances in deciphering the blueprint of neural ontogenesis and annotation of the human genome has led to the development of targeted therapeutic opportunities that have the potential of treating the most vulnerable patient populations and whose findings from benchside suggest looming clinical translation. This review discusses how findings from studies of adult neurogenesis have informed development of interventions that target endogenous neural regenerative machineries and how advances in biotechnology, including the use of new gene-editing tools, have made possible the development of promising, complex neural transplant-based strategies. Adopting a multi-pronged strategy that is tailored to underlying neural pathology and that encompasses facilitation of endogenous regeneration, correction of patient’s genomic mutations and delivery of transformed neural precursors and mature disease-relevant neuronal populations to replace injured or lost neural tissue remains no longer a fantasy.

## Introduction

Contrary to long held belief, adult neurogenesis (AN) is now a well-recognized phenomenon in mammals. While constitutively active AN is restricted to two forebrain regions, the subgranular zone (SGZ) of the dentate gyrus (DG) of the hippocampus and the subventricular zone (SVZ) lining the ventricles, variable degrees of reactive neurogenesis is present in several other brain regions that is activated in response to injury or disease onset (Jin et al., 2006). Importantly, impairments in AN have been linked to a number of neurological and psychiatric diseases (Hoglinger et al., 2004; Reif et al., 2006), indicating the value of developing therapies that boost AN potential of cell replacement in tackling brain dysfunction.

In contrast to mammals, lower vertebrates such as teleost fish and amphibians (such as axolotl and salamander) have expanded neurogenic capacities. Unsurprisingly, most of our understanding of human AN processes continues to be informed by findings in lower vertebrate models that also display comparable expression patterns and functions of neurogenic molecular controls. Intriguingly, some amphibian species display life-long de-differentiation and *trans-*differentiation processes during repair (as seen during retinal and lens regeneration in newts), phenomenon absent in mammals. Using recently developed gene-editing toolkits, studies in these species can provide mechanistic insights into why such phenomenon are scaled down in mammals and be informative for developing novel disease relevant cell-replacement strategies.

**FIGURE 1:**
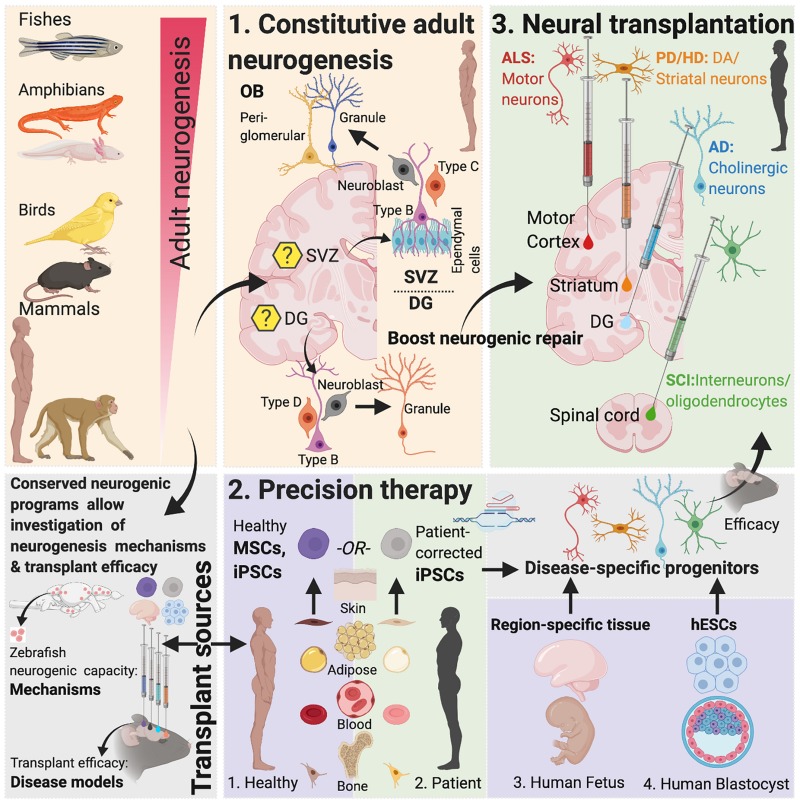
Adult neurogenesis in mammals and non-mammals. Lessons from non-vertebrate neurogenesis and implications for designing cell-based transplant therapies. Mammalian SVZ Neurogenesis: Characterization of neurogenesis in the rodent SVZ has revealed the existence of quiescent and active populations of proliferating cells. While heterogenous populations of NSCs have been described, “type-B” cells maintaining close proximity with the ependymal cell layer include nestin-expressing populations that asymmetrically divide to form Ascli and Dlx2 expressing “type-C” cells, also called transit-amplifying cells, which then symmetrically divide to form Dcx-positive type-A neuroblasts. These neuroblasts constitute the rostral migratory stream (RMS) that eventually contributes to OB peri-glomerular and granule cells. Recent studies have suggested that “type-B” cells are derived from embryonic NSCs that also generate striatal, septal or cortical neurons and become quiescent between E13.5-15.5 until their activation in adulthood (Fuentealba et al., 2015). Within the OB, granule cells form >95% of the adult-born population (Winner et al., 2002; Naritsuka et al., 2009; Merkle et al., 2014). Optogenetic activation paired with odor stimulation of adult-born neurons has been shown to facilitate difficult olfactory discrimination learning, an effect that is absent following photoactivation of early postnatal born neurons (Alonso et al., 2012). In addition, two photon-targeted recordings from peri-glomerular neurons have revealed that adult-born cells functionally integrate in the OB circuitry and whose activity is regulated by experience-dependent plasticity (Livneh et al., 2014). Mammalian DG Neurogenesis: Whether the adult mammalian hippocampus contains self-renewing NSCs or whether the neighboring lateral ventricular niche containing true NSCs maintains neurogenesis in the hippocampus has been contested (Seaberg and van der Kooy, 2002; Bull and Bartlett, 2005) but several recent studies have documented the presence of self-renewing, multipotent cells expressing embryonic NSC markers in the DG with fate mapping analysis confirming their stem cell behavior *in vivo* (Seri et al., 2001; Lagace et al., 2007; Imayoshi et al., 2008; Lugert et al., 2010; Bonaguidi et al., 2011; Encinas et al., 2011; Urban et al., 2016; Pilz et al., 2018). In the adult hippocampus (DG), SGZ radial glia-like cells give rise to proliferative non-radial-like populations that differentiate into neuroblasts. Upon differentiation, these neuroblasts develop into granule cells (Pilz et al., 2018). The embryonic origin of adult precursors in the DG in rodents was recently traced to a common Hopx-positive precursor population that is responsible for generating both developmental and AN, with the precursors not undergoing a lineage specification change during any stage of development (Berg et al., 2019). Non-mammalian Vertebrates: In contrast to mammals, non-mammalian vertebrates including certain fish and salamander species display a remarkable amount of neurogenic capacity in the adult. More than a dozen constitutive neurogenic zones have been described in the adult teleost (Grandel et al., 2006; Maruska et al., 2012), and while constitutive neurogenesis is limited to the forebrain in the newt (Berg et al., 2010) and the forebrain and ventricular zone in the axolotl (Maden et al., 2013), neuronal loss in these species following injury leads to complete regeneration (Bernardos et al., 2007; Parish et al., 2007; Berg et al., 2010; Skaggs et al., 2014). This latter capacity is reminiscent of, albeit less efficacious, reparative recruitment of quiescent progenitors found in rodents (Barnabe-Heider et al., 2010; Sirko et al., 2013; Magnusson et al., 2014). Implications for Designing Brain Repair Strategies: The limited ability of AN in mammals to enable neural repair could be due to a presence of reduced number of NSCs, available progenitors that maybe fate-restricted or strong inhibitory cues from glia. As mentioned, studies focused on naturally occurring neurogenic process that are present in lower vertebrates can yield important insights into why those mechanisms are restricted in mammals (Bhattarai et al., 2016, 2020). Evidence for the presence of evolutionary conserved genes related to regeneration as revealed by recent genome sequencing of species with enhanced regenerative capacities and the development of techniques that make those species amenable to gene-editing (Howe et al., 2013; Irion et al., 2014; Elewa et al., 2017; Nowoshilow et al., 2018) has paved the way for in-depth investigation of neurogenic molecular controls and their comparison across mammals and non-mammalian species to design novel therapies. Created with Biorender.com.

The remarkable ability of the adult mammalian brain to functionally integrate new neurons into existing circuitries (Falkner et al., 2016) combined with the progress in our understanding of these phenomenon in non-mammalian and mammalian models has now made possible the development of therapies that can awaken latent neurogenic programs, direct resident neuronal fates into cell types of need and replace injured or diseased neurons in brain regions within and outside that of DG and SVZ. This review discusses AN in mammalian and non-mammalian brains, the reparative potential of cell-based transplant therapy in neurodegenerative diseases and the therapeutic application of novel gene-editing approaches in the framework of designing disease- and patient-specific curative strategies (Figure 1).

## Adult Neurogenesis in the Mammalian Brain

### Evidence of Adult Neurogenesis in Mammals: A Brief History

Initial anatomical evidence of the existence of AN in mammals did not arrive until the 1960s when, using thymidine-H^3^ autoradiographic techniques, Joseph Altman reported evidence of the formation of newborn neurons in the adult brain (Altman, 1962, 1963; Altman and Das, 1965, 1967). Follow-up electron microscopic studies extended support to the claim of radio-labeled cells as being neuronal (Kaplan and Hinds, 1977), still, a lack of use of definitive neuronal markers to co-label radio-labeled cells and a failure to replicate this in non-human primates (Rakic, 1985; Eckenhoff and Rakic, 1988) prevented the field from reaching consensus.

Almost a decade after Altman’s initial findings, Fernando Nottebohm, following up on his discovery of sexually dimorphic and seasonally regulated song-control system in the songbird (Nottebohm and Arnold, 1976; Nottebohm, 1981), crucially demonstrated adult-born cells’ neuronal identity and functional integration into adult circuits (Goldman and Nottebohm, 1983; Paton and Nottebohm, 1984; Alvarez-Buylla et al., 1990a). Soon after, the glial nature of NSCs in songbirds was described (Alvarez-Buylla et al., 1990b), which was followed by similar observations in the mammalian SVZ (Doetsch et al., 1999) and hippocampus (Seri et al., 2001, 2004). Later, the isolation of mitogen-responsive multipotent cells and successful induction of reactive neurogenesis suggested continuance of prenatal permissive regenerative programs into the adult mammalian brain (Reynolds and Weiss, 1992; Lois and Alvarez-Buylla, 1993; Luskin, 1993; Palmer et al., 1995). Finally, AN in the SVZ and hippocampus was shown to exist in humans (Eriksson et al., 1998; Spalding et al., 2013; Ernst et al., 2014).

#### Adult Neurogenesis in the Sub-Ventricular Zone

Initial characterization of the adult human SVZ by Alvarez-Buylla et al., reported a ribbon of astrocytes lining the lateral ventricles with few surrounding proliferating cells (Sanai et al., 2004). In particular, the human SVZ was reported to lack the cellular organizational structure and rostral migratory stream (RMS) characteristics of its rodent counterpart (Figure 1, legend). A follow-up study by the same group reported strong expression of immature cell markers and RMS in the infant human SVZ, both of which sharply declined after birth (Sanai et al., 2011). In addition, a novel migratory stream to the cortex was described in the infant brain (Sanai et al., 2011). In contrast, another group found robust SVZ proliferation and a RMS containing glial cells, proliferating cells and neuroblasts organized around a lateral ventricular extension reaching the olfactory bulb (OB) in the adult (Curtis et al., 2007; Kam et al., 2009). These disparate adult human data underscore the importance of applying more reliable, species-specific progenitor/immature cell markers and optimizing methodological approaches for identifying NSCs (see also below). From a therapeutic perspective, the existence of SVZ neurogenesis in humans could have important implications for neurodegenerative disorders as neuroblasts were recently shown to migrate to and differentiate into interneurons in the striatum (Ernst et al., 2014) [but also see Dennis et al. (2016)] and carbon-14 birth-dating studies have suggested that adult-born striatal neurons might be preferentially depleted in Huntington’s disease (HD) (Ernst et al., 2014). Moreover, in animals models of PD, stimulation of SVZ precursors has been shown to rescue dopamine (DA) mediated behaviors (Spalding et al., 2005; Androutsellis-Theotokis et al., 2009).

#### Adult Neurogenesis in the Hippocampal Dentate Gyrus

Whether the adult human hippocampus contains self-renewing NSCs is a topic of much interest. Eriksson et al. (1998) initially reported newly generated BrdU-labeled cells in the human DG that co-expressed neuronal markers. This finding was corroborated by studies demonstrating the presence of neural progenitors in surgically excised brain specimens (Roy et al., 2000; Palmer et al., 2001) and carbon-14 birth-dating studies that revealed substantial DG neuronal turnover (Spalding et al., 2013). Recently, Boldrini et al. (2018) also found preserved DG neurogenesis in individuals 14–79 years of age, with older individuals having a smaller quiescent progenitor pool. The existence of lifelong hippocampal AN was further confirmed in two very recent studies that examined neurogenesis throughout normal and pathological aging in AD patients (Moreno-Jimenez et al., 2019) and in individuals with mild cognitive impairment (Tobin et al., 2019), with findings suggesting impaired neurogenesis in both conditions. This is not surprising since an enhanced DG neurogenic capacity has been linked to reduced susceptibility to cognitive impairments in patients with AD pathology (Briley et al., 2016) and enhancing hippocampal neurogenesis improved cognition in an AD mouse model (Choi et al., 2018). In stark contrast, other groups have observed a sharp decline in proliferating cells in childhood, observing only sparse number of proliferating cells in the adult hippocampus that were of microglia lineage (Dennis et al., 2016). In addition, Sorrells et al. (2018), using DCX and PSA-NCAM to label immature neurons, found a sharp reduction in hippocampal neurogenesis in childhood observing no new neurons in the adult. Intriguingly, the authors found that both these markers, which are widely used to identify neurogenesis in other species, can label mature neurons and glial cells in humans. Marker expression can depend on tissue preservation techniques and DCX is known to be sensitive to postmortem breakdown (Boekhoorn et al., 2006) posing significant challenges to cross-study comparisons. Future studies could benefit by allocating efforts to documenting the clinical profile and mode of death of patients as both these factors can drastically influence neurogenesis rates. In addition, applying quality control procedures, including minimizing postmortem delay, optimizing brain fixation methods to preserve neuronal morphology and marker expression, standardization of protocols for identification of neurogenic neurons and examination of gene expression patterns, along with development of more specific markers of neurogenic neurons in humans may be beneficial.

### Adult Neurogenesis in the Non-mammalian Brain: Similarities to Adult Mammalian Neurogenesis

Several similarities exist between mammalian and non-mammalian species regarding AN. These include the sites of neurogenesis [the ventricular lining within the telencephalon appears to be the site of origin of new neurons in many mammalian and non-mammalian species (Goldman and Nottebohm, 1983; Doetsch et al., 1999; Ganz et al., 2010; Rothenaigner et al., 2011)], identity of neurogenic progenitors as well as the molecular machineries that regulate proliferation of NSCs. Like in mammals (Doetsch et al., 1999), some progenitor populations in birds, fishes and amphibians also have glial characteristics (Alvarez-Buylla et al., 1990b; Rothenaigner et al., 2011; Kirkham et al., 2014). Moreover, molecular controls governing quiescence of NSCs show remarkable conservation between vertebrate species: e.g., Notch signaling has been shown to regulate radial glial quiescence in zebrafish (Alunni et al., 2013), newt (Kirkham et al., 2014) and adult mouse (Imayoshi et al., 2010) and transcription factors Id1 and Fezf2 that are associated with increased quiescence in adult mouse NSCs (Nam and Benezra, 2009) have also been identified in zebrafish (Berberoglu et al., 2014; Rodriguez Viales et al., 2015). Intriguingly, Ascl1 upregulation has been linked to the activation of the retinal latent progenitors, Muller glia, in zebrafish during retinal regeneration following lesions (Ramachandran et al., 2011) and forced overexpression of Ascl1 in Muller glia in young mice coaxes these progenitors toward a neurogenic fate (Ueki et al., 2015) rather than the gliogenic fate that is normally seen in older mice (Dyer and Cepko, 2000). Comparative studies of species can thus yield important insights into the biology of AN and be informative for therapeutic strategies that harness AN potential of tissue repair (Kizil and Bhattarai, 2018; Figure 1).

## Neural Transplantation as Cell-Based Therapy

On the other hand, transplantation of NSCs or progenitors to replace diseased neurons is an alternate strategy that comes with the opportunity of directing therapy to brain regions where it is most needed (Figure 1 and Table 1). Also, the self-renewing attribute and migratory nature of NSCs/progenitors make them attractive therapeutic candidates in the setting of diffuse damage to the brain.

**TABLE 1:** Summary of select studies involving cell-transplant based repair strategies in *in vivo* pre-clinical neurodegenerative disease models.

Repair strategy	References	Type of cell grafted/manipulated	Disease/model	Functional impact
Fetal-tissue based	(Bjorklund and Stenevi, 1979)	Fetal ventral midbrain tissue	PD/rat 6-OHDA	Restoration of DA innervation and motor improvement
	(Isacson et al., 1984)	Fetal striatal tissue	HD/rat Ibotenic-acid	Reduction in locomotor and metabolic hyperactivity
	(Low et al., 1982; Gage et al., 1984)	Fetal substantia nigra and septal nuclei	Aging, hippocampal lesions	Improved motor coordination and spatial learning
	(Gaillard et al., 2007; Falkner et al., 2016)	Fetal tissue	Motor, visual cortex	Long range, synaptic, functional integration with host circuitries
MSCs-based	(Hellmann et al., 2006)	mBM-MSCs	PD/rat 6-OHDA	Cells migrate to lesioned hemisphere and differentiate into neurons
	(Bahat-Stroomza et al., 2009)	hBM-MSCs	PD/rat 6-OHDA	Reduction in motor impairments, regeneration of DA terminals
	(Lee et al., 2009)	hAD-MSCs	HD/rat QA-lesion, mouse R6/2	Improved motor performance, reduced huntingtin aggregates
	(Lin et al., 2011)	hBM-MSCs	HD/mouse QA-lesion, R6/2	Improved motor performance in QA-lesion model
	(Shin et al., 2014)	hMSCs	AD/mouse Aβ treated	Increased autophagy and Aβ clearance
	(Garcia et al., 2014)	VEGF overexpressing BM-MSCs	AD/mouse APPswe/PS1 double transgenic	Increased vascularization, cognition, decreased Aβ plagues
	(Kim et al., 2010)	ALS-hBM-MSCs	ALS/mouse SOD1^G93A^	Increased lifespan, increased MN survival
	(Kim et al., 2014)	hAD-MSCs	ALS/mouse SOD1^G93A^	Release of growth factors, increased life span
hESCs-based	(Kriks et al., 2011)	hESC-DA neurons	PD/mouse, rat 6-OHDA, monkey MPTP	Long term survival and motor restoration
	(Grealish et al., 2014)	hESC-DA neurons	PD/rat 6-OHDA	Motor restoration comparable to human fetal grafts
	(Steinbeck et al., 2015)	Inhibitory opsin-expressing hESC-DA neurons	PD/mouse 6-OHDA	Light-induced silencing of grafts re-introduced motor defects
	(Chen et al., 2016)	CRISPR-engineered DREADD expressing hESC-DA neurons	PD/mouse 6-OHDA	Control of motor behaviors by CNO
	(Aubry et al., 2008)	hESC-striatal progenitors	HD/rat QA-lesion	DARPP32 + differentiation
	(Ma et al., 2012)	hESC-striatal progenitors	HD/mouse QA-lesion	Correction of locomotive deficits and circuit integration
	(Faedo et al., 2017)	hESC-striatal progenitors	HD/rat QA-lesion	Long-range circuit integration
	(Espuny-Camacho et al., 2017)	hESC-cortical progenitors	AD/chimeric APP/PS1 mouse	Susceptibility of human neurons to Tau
	(Yue et al., 2015)	hESC-basal forebrain cholinergic neurons	AD/mouse 5XFAD, APP/PS1	Improvement in learning and memory
	(Rossi et al., 2010)	hESC-MNP	SCI/rat	Improvement in motor function
	(Wyatt et al., 2011)	hESC-MNP	ALS, SMA, SCI	Increased growth factor secretion
iPSCs-based	(Hargus et al., 2010)	DA neurons differentiated from patient fibroblasts	PD/rat 6-OHDA	Correction of AMPH-induced rotation behavior
	(Samata et al., 2016)	DA progenitors differentiated from human PSCs	PD/rat 6-OHDA, monkey MPTP	Restoration of motor deficits
	(Kikuchi et al., 2017a)	DA neurons differentiated from healthy and PD fibroblasts	PD/monkey MPTP	Long-term survival of DA cells. Increase in spontaneous movement.
	(Kikuchi et al., 2017b)	Healthy or idiopathic PD-iPSCs differentiated from fibroblasts and peripheral blood cells	PD/mouse α-Synuclein, rat 6-OHDA	Lack of α-Synuclein accumulation. Motor improvement
	(Jeon et al., 2012)	CAG-repeat HD-iPSCs	HD/rat QA-lesion	Initial behavioral recovery. Development of HD pathology.
	(An et al., 2012)	CAG-repeat-corrected HD-iPSCs	HD/mouse R6/2	Rescue of pathogenic HD signaling
	(Mu et al., 2014)	Mouse iPSCs	HD/rat QA-lesion	Improved learning and memory
	(Cha et al., 2017)	Mouse iPSCs	AD/mouse 5XFAD	Reduced Aβ plaque, improved cognition
	(Fujiwara et al., 2013)	hiPSCs- cholinergic neurons	AD/mouse PDAPP	Improved spatial memory
	(Popescu et al., 2013)	hiPSCs-MNP	ALS/rat SOD1^G93A^	Motor neuron generation
	(Nizzardo et al., 2014)	hiPSCs-NSC	ALS/mouse SOD1^G93A^	Improved neuromuscular function, reduced motor neuron loss
*In vivo* direct reprogramming	(Torper et al., 2013)	Exogenous human astrocytes. Endogenous mouse striatal astrocytes	PD/mouse 6-OHDA	Conversion into DA neurons
	(Rivetti di Val Cervo et al., 2017)	Endogenous mouse striatal astrocytes	PD/mouse 6-OHDA	Conversion into DA neurons. Correction of gait
	(Pereira et al., 2017)	Endogenous midbrain and striatal NG2 glia	PD/mouse 6-OHDA	Conversion into PV neurons
	(Niu et al., 2013)	Endogenous striatal astrocytes	Aging/mouse	Conversion into neuroblasts and mature neurons
	(Guo et al., 2014)	Endogenous cortical astrocytes and NG2 glia	AD, Stab-injury/mouse	Conversion into glutamatergic and GABA neurons
	(Su et al., 2014)	Endogenous and exogenous astrocytes	SCI/T8 hemi-section mouse	Neurogenesis and conversion into GABA neurons
	(El Waly et al., 2018)	Endogenous mouse neuroblasts	Demyelination/cuprizone-induced	Conversion into myelin producing oligodendrocytes
	(Torper et al., 2015)	Endogenous mouse NG2 glia	Mouse	Conversion into glutamatergic and GABA neurons

*MSC, Mesenchymal Stem Cell; hESC, human Embryonic Stem Cell; hiPSC, human induced Pluripotent Stem Cell; PD, Parkinson’s Disease; 6-OHDA, 6-hydroxydopamine; MPTP, 1-methyl-4-phenyl-1,2,3,6-tetrahydropyridine; HD, Huntington Disease; QA, Quinolinic Acid; R6/2, Huntingtin transgene insertion 62; hBM, human bone marrow; hAD, human Adipose; Aβ, beta-amyloid; AD, Alzheimer’s Disease; APPswe/PS1, amyloid beta precursor protein/presenilin 1; 5XFAD, mice express human APP and PSN1 transgenes with a total of five AD-linked mutations; PDAPP, APP expressed under PDGF promoter; ALS, amyotrophic lateral sclerosis; SOD1, superoxide dismutase 1; SCI, spinal cord injury; SMA, spinal muscular atrophy.*

### Fetal and Embryonic Tissue-Based Transplants in Pre-clinical Models and Clinical Trials

Initial proof-of-concept transplantation studies carried out during 70 and 80s demonstrated long-term viability of grafted neural tissue and evidence for functional replacement for missing neurons. In animal models of PD and HD, transplanted fetal rat substantia nigra tissue established connections with host striatum and reversed motor and metabolic deficits (Perlow et al., 1979; Isacson et al., 1984). Additional support came from the long-term survival of human fetal neural tissue in monkeys (Redmond et al., 1988), that led to the commencement of transplantation of fetal tissue into the striatum of PD patients (Lindvall et al., 1990; Spencer et al., 1992). Intriguingly, the initial open-label clinical trials demonstrated restoration of DA synthesis resulting in significant and long-lasting clinical improvements of motor function (Hauser et al., 1999; Piccini et al., 1999). Though variable clinical efficacy was observed, the ability of allografts to survive, integrate and function in diseased host environment, in some cases up to 20 years (Bega and Krainc, 2014), was in itself an exciting finding. Subsequent double-blinded, placebo controlled clinical trials, however, failed to reveal definitive results and long-term follow up studies revealed the development of α-synuclein aggregates in grafted fetal neurons (Kordower et al., 2008) and graft-induced dyskinesias (Freed et al., 2001; Hagell et al., 2002; Olanow et al., 2003), effects later attributed to heterogenous spread of transplanted cells and/or graft contamination by serotonergic cells (Politis et al., 2010). In addition, early withdrawal of immunosuppression, severe phenotype of transplanted patients and grafts’ non-innervation of the ventral striatum were identified as some of the limiting factors. Based on these findings, a new multi-center trial with fetal-based transplantation to PD patients is currently underway that hopes to address these concerns (Moore et al., 2014; Kirkeby et al., 2017). Therapeutic potential of neural transplantation was similarly first explored for HD in rodent and non-human primate models (Wictorin et al., 1989; Peschanski et al., 1995; Kendall et al., 1998), the positive results of which led to fetal striatal implantation in patients in the mid- to late-90s (Bachoud-Levi et al., 2000, 2006; Hauser et al., 2002). However, disease-like neuronal degeneration of grafted tissue upon postmortem analysis raised uncertainty about this approach (Cicchetti et al., 2009; Table 1).

### Limitations of Using Fetal and Embryonic Origin Cell-Based Transplantation Strategies

It is worth mentioning here that both PD and HD, at least in their earlier stages, involve relatively specific cellular and regional pathologies: nigrostriatal DA neurons in PD and striatal medium spiny neurons in HD, making them suitable to tissue grafts dissected from fetal regions that contain the target cell population. However, it should also be kept in mind that pathologies in these conditions are not region-specific. For instance, in PD, non-DA and non-motor symptoms can cause significant disability, especially in patients with advanced pathology (Chaudhuri et al., 2006) and while striatal pathology dominates initial stages of HD, cell loss in the motor and cingulate cortices correlates with the degree of motor and mood dysfunction characteristic of the disease in later stages (Thu et al., 2010). Therefore, to alleviate the full repertoire of advanced disease symptomatology, adjuvant therapy for secondary disease processes maybe required. Indeed, widespread brain degenerative changes have been proposed to offset therapeutic efficacy of an otherwise viable graft (Li et al., 2016). Such a strategy may also not be applicable to disorders of diffuse pathology such as stroke, AD, SCI, and ALS, that involve pathological alterations in multiple neuronal and glial cellular phenotypes and brain regions. Lastly, the most obvious limitation to fetal transplants is the limited availability of graft sources which have traditionally been derived from human embryos causing significant logistical and ethical concerns.

## Precision Therapeutics

### Human Embryonic and Induced Pluripotent Stem Cell Technologies

Given the variable clinical efficacy, risk of contamination by non-specific cell types and ethical concerns associated with fetal grafts, alternative transplant sources have been sought to boost endogenous reparative mechanisms. Recently, pluripotent stem cells (PSCs) (Gurdon, 1962; Evans and Kaufman, 1981; Martin, 1981) including hESCs (Thomson et al., 1998; Reubinoff et al., 2000) derived from pre-implantation embryos, and hiPSCs (Takahashi and Yamanaka, 2006; Yu et al., 2007; Park et al., 2008) that are reprogrammed from somatic cells using a defined cocktail of transcription factors, have attracted tremendous interest in the field of regenerative medicine. Use of autologous hiPSCs can also overcome immune mismatch-mediated graft rejection and circumvent ethical and logistical issues associated with the use of hESCs. While hiPSC technology is by design usable to treat donor patients limiting its cost-effectiveness and scalability (Nakatsuji et al., 2008), hypo-immunogenicity in hiPSCs was recently achieved via inactivation of the major histocompatibility complex (MHC) and overexpression of the transmembrane protein CD47 (Deuse et al., 2019), potentially conferring a remarkable universal donor capability to hiPSCs (Figure 1 and Table 1).

### Genome-Editing

The development of biotechnology allowing generation of hiPSCs from non-viral techniques and gene-editing tools that enable site-specific corrections of disease-causing gene mutations have made genome-edited hiPSC-based cell therapy an ideal choice in the field of precision therapeutics. Using genome-editing tools, such as zinc finger nucleases (ZFNs) (Bibikova et al., 2003), TAL effector nucleases (Boch et al., 2009) and the more specific clustered regulatory interspaced short palindromic repeats (CRISPR)/CRISPR-associated (Cas) system (Mali et al., 2013), that enable precise corrections of deleterious mutations, genome corrected-iPSCs are now being generated. Already, advances have led to generation of engineered cells that are able to deliver therapeutic factors and that carry minimal risks of tumorigenicity in preclinical models of PD (Kriks et al., 2011; Grealish et al., 2014; Hallett et al., 2015; Steinbeck et al., 2015; Kikuchi et al., 2017a; Shi et al., 2017). Using ZFNs, generation of isogenic disease and control hiPSCs from PD patients’ cortical neurons carrying the α-synuclein mutations A53T (Chung et al., 2013) and LRRK2 (Reinhardt et al., 2013) and their insertion into hESCs (Soldner et al., 2011) have been described. Lately, CRISPR/Cas system has been used to correct α-synuclein mutations in PD hiPSCs (Heman-Ackah et al., 2016; Soldner et al., 2016), remove HTT-repeat expansion mutation in HD hiPSCs (Xu et al., 2017) and correct mutations in presenilin (PSEN) in AD basal forebrain cholinergic neurons (Ortiz-Virumbrales et al., 2017). While majority of these studies have employed *ex vivo* manipulation of hiPSCs, successful *in vivo* gene editing of post mitotic cells, as has been shown in five-familial AD and amyloid precursor protein (APP) knock-in mice (Park et al., 2019), has opened up the tantalizing prospect of eliminating the need for cell transplants for brain repair. In theory, *in vivo* gene editing is a similar concept to the one used for *in vivo* direct reprogramming in which non-neuronal cells are converted into disease-specific neurons directly *in situ* with preclinical studies showing successful integration of reprogrammed cells into host circuits within the cerebral cortex (Guo et al., 2014), striatum (Torper et al., 2015), midbrain (Torper et al., 2013) and the spinal cord (Su et al., 2014) in heterogenous disease contexts (Figure 1 and Table 1).

## Discussion

### Applying a Multi-Pronged Strategy for Repair of Neural Tissue

Alterations in AN appear to be a common feature in many neurological and psychiatric disorders (Sahay and Hen, 2007; Winner et al., 2011; Niv et al., 2012; Pun et al., 2012). Studies in experimental models can yield important insights into AN biology including its potential for cell replacement in disease and injury contexts (van Tijn et al., 2011; Newman et al., 2014). Yet to achieve successful tissue restoration, a combinatorial therapeutic strategy might be required that includes boosting of endogenous neurogenic processes, *in vivo* genome-correction of endogenously generated NSCs/progenitors/mature neurons, transplantation of *ex vivo* gene-corrected neurons and the delivery of neurotherapeutic factors. Transplantation of fate-restricted progenitors that retain the capability to migrate and differentiate into mature neurons and functionally integrate into existing circuits has the benefit of decreasing the number of transplants and chances of developing non-specificity issues. Still, multiple cellular phenotypes might be required to construct a therapeutically efficacious graft, whose composition must be geared toward correcting primary genetic deficits and secondary phenotypes. Transplant composition for chronic neurodegenerative diseases, especially in advanced stages, might require a greater dependence on exogenous cells due to the extensive damage to host tissue, while also attempting to correct mutations in remaining diseased neuronal populations. Acute injuries, on the other hand, would require a greater role for neurotrophic factors and modification of extracellular matrix to facilitate regeneration of existing tissue, while at the same time replacing lost or damaged neural populations by promoting endogenous neurogenesis and transplanting fate-restricted precursors and/or reprogrammed mature neurons. Given the above, perhaps the most effective approach while designing cell-based therapy might be to adopt a multi-pronged strategy that cumulatively addresses the shortcomings of the above-mentioned tissue restoration strategies and that is tailored to the brain disease in hand.

## Author Contributions

MC developed the concepts and wrote the manuscript.

## Conflict of Interest

The author declares that the research was conducted in the absence of any commercial or financial relationships that could be construed as a potential conflict of interest.
